# Protective Effects of Tormentic Acid on Unilateral Ureteral Obstruction-Induced Renal Injury, Inflammation, and Fibrosis: A Comprehensive Approach to Reducing Oxidative Stress, Apoptosis, and Ferroptosis

**DOI:** 10.3390/antiox14010013

**Published:** 2024-12-25

**Authors:** Ah Young Yang, Jung-Yeon Kim, Mi-Gyeong Gwon, Hyun Hee Kwon, Jaechan Leem, Eon-Ju Jeon

**Affiliations:** 1 Department of Immunology, School of Medicine, Daegu Catholic University, Daegu 42472, Republic of Korea; diddkdud123@naver.com (A.Y.Y.); jy1118@cu.ac.kr (J.-Y.K.); daldy99@naver.com (M.-G.G.); 2 Department of Internal Medicine, School of Medicine, Daegu Catholic University, Daegu 42472, Republic of Korea; heeya0035@cu.ac.kr

**Keywords:** tormentic acid, renal fibrosis, oxidative stress, apoptosis, ferroptosis

## Abstract

Chronic kidney disease (CKD) progresses through mechanisms involving inflammation, fibrosis, and oxidative stress, leading to the gradual structural and functional deterioration of the kidneys. Tormentic acid (TA), a triterpenoid compound with known anti-inflammatory and antioxidant properties, shows significant potential in counteracting these pathological processes. This study explored the protective role of TA in a unilateral ureteral obstruction (UUO)-induced CKD model. Mice received TA through intraperitoneal injections at a dosage of 5 mg/kg per day for 8 consecutive days, commencing a day before the UUO procedure. The TA treatment significantly improved both structural and functional kidney injury. It suppressed cytokine expression and reduced immune cell infiltration, inhibited the activation of the mitogen-activated protein kinase cascade, and alleviated endoplasmic reticulum stress. Moreover, TA displayed potent anti-fibrotic effects by reversing epithelial-to-mesenchymal transition and inhibiting Smad2/3 activation, reducing extracellular matrix deposition. TA also mitigated oxidative stress by attenuating lipid peroxidation and boosting antioxidant defenses. Additionally, it inhibited apoptosis and ferroptosis by reducing oxidative stress and modulating key cell death markers. Collectively, these findings indicate that TA provides comprehensive renoprotection in the UUO model by effectively targeting inflammation, fibrosis, oxidative stress, and tubular cell death in CKD progression.

## 1. Introduction

Chronic kidney disease (CKD), defined as the progressive decline in renal function, is a significant global health challenge [1]. It often progresses silently, with few symptoms until significant damage has occurred. In its advanced stage, critical interventions like dialysis or organ transplantation become essential to maintain life [1]. The mechanisms of renal injury in CKD involve a complex interplay between inflammation, oxidative stress, and fibrotic processes, which collectively cause the continuous deterioration of renal function over time [2,3]. Current therapeutic options primarily focus on managing symptoms and slowing disease progression. However, they do not address the root causes of CKD comprehensively. There remains an urgent need for novel treatments that can target these underlying mechanisms more effectively, aiming to prevent, halt, or even reverse renal damage.

Tubular cell death is a critical factor in driving renal inflammation and fibrosis, with apoptosis and ferroptosis being key processes involved [4,5]. Apoptosis leads to the loss of functional tubular cells, which in turn triggers inflammatory responses and fibrosis through the secretion of various cytokines that recruit immune cells and stimulate fibrotic pathways [5]. Oxidative stress plays a pivotal role in inducing apoptosis in CKD, as it causes cellular damage that triggers the apoptotic cascade, ultimately contributing to heightened inflammation and fibrosis [2,3]. Ferroptosis, an iron-dependent form of cell death characterized by lipid peroxidation, amplifies tubular damage and plays a significant role in advancing renal fibrosis [6]. Ferroptosis is particularly important in CKD due to its association with heightened oxidative stress and iron dysregulation, both of which are prevalent in CKD. Controlling these cell death mechanisms is crucial, as their direct impact on inflammation and fibrosis makes them key therapeutic targets for mitigating renal injury and slowing CKD progression. Studies have shown that the inhibition of apoptosis and ferroptosis can reduce renal inflammation and fibrosis [7,8,9,10], underscoring the need for effective interventions targeting these pathways. Therefore, novel therapeutic strategies that target oxidative stress, apoptosis, and ferroptosis are essential to effectively manage CKD and prevent irreversible renal damage.

Tormentic acid (TA), a triterpenoid derived from several medicinal plants, is known for its diverse therapeutic activities, such as reducing inflammation, combating oxidative stress, inhibiting microbial growth, and exerting anticancer effects [11]. Previous animal studies have demonstrated that TA exerts protective effects against neurodegenerative diseases [12,13], acetaminophen-induced hepatotoxicity [14], and diabetes [15]. TA exerts its protective effects through its ability to scavenge free radicals, inhibit inflammatory signaling pathways, and modulate cellular redox status, thereby reducing oxidative stress and cellular injury [11]. Recent studies have also suggested its potential in modulating cell death pathways in non-cancer cells [16,17,18]. By inhibiting apoptosis and ferroptosis, TA may help reduce tubular cell death, inflammation, and fibrosis in CKD. Given these properties, TA may have potential as a therapeutic candidate for mitigating CKD by targeting key mechanisms involved in disease progression.

This study investigates the protective effects of TA in a unilateral ureteral obstruction (UUO)-induced CKD model, with a focus on its ability to reduce inflammation, fibrosis, oxidative stress, apoptosis, and ferroptosis. The UUO model is widely recognized for studying progressive renal injury, including inflammation and fibrosis [19,20]. By thoroughly examining the mechanisms through which TA mitigates these pathological processes, this study aims to enhance our understanding of TA’s multifaceted effects and its potential as a protective agent in CKD.

## 2. Materials and Methods

### 2.1. Animal Experiments

**Figure 1:**
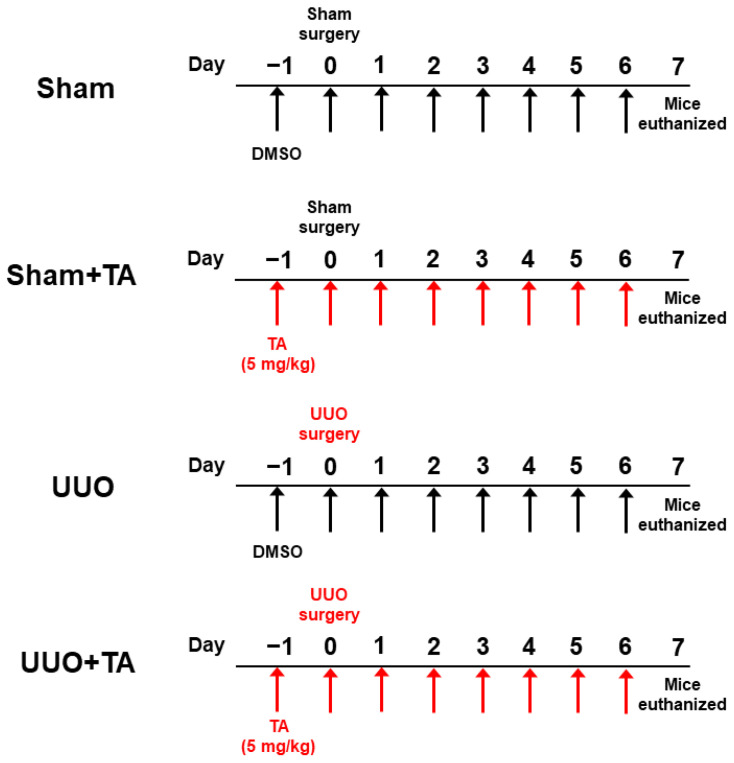
Experimental design for TA treatment in UUO-induced CKD mouse model.

The experimental design received approval from the Institutional Animal Care and Use Committee of the Daegu Catholic University Medical Center (DCIAFCR-230615-15-Y), ensuring compliance with ethical standards. Male C57BL/6J mice, aged six weeks, were acquired from Samtako (Osan, Republic of Korea). The mice were housed under regulated conditions, maintaining a temperature of 20–24 °C and a 12 h alternating light and dark cycle. After a one-week adaptation period, the mice were arbitrarily divided into four experimental groups (Figure 1), with each group comprising 8 mice: (1) a control group undergoing a sham operation without any treatment (sham), (2) a group undergoing a sham operation followed by TA treatment (sham + TA), (3) a group subjected to UUO surgery (UUO), and (4) a group receiving UUO surgery followed by TA treatment (UUO + TA). The UUO model was induced by accessing the left kidney through a lateral incision while the mouse was under general anesthesia. The left ureter was carefully isolated and tied at two distinct sites with 5-0 silk sutures, leaving the segment between the ties intact to create a complete obstruction. In the sham-operated groups, an identical surgical procedure was conducted, including kidney exposure, but without ligating the ureter. In the TA-treated groups, mice received daily intraperitoneal injections of TA (Cayman Chemical, Ann Arbor, MI, USA) at a dosage of 5 mg/kg (prepared in DMSO) over a period of 8 days, beginning one day before surgery. The sham and UUO groups were administered daily intraperitoneal injections containing the same volume of DMSO as a vehicle control. The TA dosage was selected based on its previously reported efficacy in other studies [13,14]. One week after surgery, all mice were subjected to anesthesia and subsequently euthanized.

### 2.2. Biochemical Measurements

BUN and creatinine concentrations in serum were quantified with a Hitachi automated system (Osaka, Japan). Renal concentrations of TNF-α, IL-6, and IL-1β were measured using immunoassay kits from R&D Systems (Minneapolis, MN, USA). Renal tissue myeloperoxidase (MPO) activity was measured using a kit from Abcam (Cambridge, MA, USA). Malondialdehyde (MDA) concentrations in renal tissue were assessed with a detection kit from Sigma-Aldrich (St. Louis, MO, USA). Glutathione (GSH) concentrations in renal tissue were evaluated using an assay kit provided by Enzo Life Sciences (Farmingdale, NY, USA).

### 2.3. Histological Evaluation and Immunohistochemical (IHC) Analysis

Tissue samples were carefully collected, and after fixation in 4% paraformaldehyde, they underwent gradual dehydration using increasing concentrations of ethanol. Subsequently, the tissues were embedded in paraffin blocks. To evaluate renal structure, PAS staining was used, while Masson’s trichrome staining was employed to assess fibrosis. Renal injury was scored by examining pathological changes in cortical areas from 10 randomly selected fields (400×) per sample, using a scale from 0 to 4; a score of 0 indicated no injury (0%), 1 represented mild injury (≤25%), 2 indicated moderate injury (25–50%), 3 indicated severe injury (50–75%), and 4 represented extensive damage (>75%) [21]. IHC staining was performed by removing paraffin from the tissue sections using xylene, followed by gradual rehydration through descending concentrations of ethanol. Antigen retrieval was carried out through heat treatment to enhance antibody binding. The sections were treated with primary antibodies targeting F4/80 and 4-hydroxynonenal (4-HNE). The F4/80 antibody was obtained from Santa Cruz Biotechnology (Santa Cruz, CA, USA), and the 4-HNE antibody was acquired from Abcam (Cambridge, MA, USA). After the primary antibody treatment, the samples were rinsed and treated with secondary antibodies linked to horseradish peroxidase (HRP), chosen according to the host species of the primary antibodies. Signal detection was performed using diaminobenzidine (DAB) as the chromogen, followed by counterstaining with hematoxylin. High-resolution visual data of the stained sections were obtained using a digital slide scanner (3DHISTECH Pannoramic MIDI, Budapest, Hungary). Masson’s trichrome and 4-HNE signals were quantified using image analysis software version 11.0 (IMT i-Solution, Coquitlam, BC, Canada) in 10 randomly selected cortical fields (200×) for each specimen. F4/80-stained cell counts were manually performed in 10 arbitrarily chosen cortical fields (600×) for each specimen.

### 2.4. Immunofluorescent (IF) Staining

Tissue sections were treated with primary antibodies against Ly6B.2 (Abcam, Cambridge, MA, USA) and 8-OHdG (Bioss, Woburn, MA, USA), after which the corresponding secondary antibodies were applied. To identify the brush border in proximal tubules, FITC-conjugated lotus tetragonolobus lectin (LTL) was applied, which was purchased from Vector Laboratories (Burlingame, CA, USA). DAPI was used for nuclear counterstaining. Isotype control antibodies were used as negative controls for Ly6B.2 and 8-OHdG staining, and a no primary antibody control was applied for FITC-conjugated LTL staining. LTL-stained areas were evaluated using image analysis software version 11.0 (IMT i-Solution, Coquitlam, BC, Canada) on randomly selected cortical fields (400×) per sample. For Ly6B.2-positive and 8-OHdG-positive cells, manual counting was conducted in 10 randomly selected cortical fields (600×) for each specimen.

### 2.5. TUNEL Staining

To identify apoptosis in tissue samples, a TUNEL staining kit from Roche Diagnostics (Indianapolis, IN, USA) was used. Tissue sections were deparaffinized using xylene washes, then rehydrated through ethanol and water. Permeabilization was achieved by treating sections with Proteinase K at 37 °C for 30 min, followed by a PBS rinse to remove residual enzymes. After equilibration in the provided buffer, the TdT-mediated reaction mixture (TdT enzyme with labeled dUTP) was applied to each section, allowing labeled nucleotides to bind to DNA fragments. For the negative control, the same procedure was followed, but the TdT enzyme was omitted from the reaction mixture to ensure the specificity of the TUNEL staining. The nuclei were stained with DAPI. TUNEL-positive cells were identified as green-fluorescent nuclei and counted in 10 arbitrarily selected fields (600×) for each specimen.

### 2.6. Western Blotting

To extract protein from tissue samples, the tissues were homogenized in a lysis buffer. The protein samples were separated using SDS-PAGE on polyacrylamide gels. After electrophoresis, proteins were transferred onto nitrocellulose membranes. The membranes were then probed with primary antibodies targeting a range of proteins, including neutrophil gelatinase-associated lipocalin (NGAL; Santa Cruz Biotechnology, Santa Cruz, CA, USA), glyceraldehyde-3-phosphate dehydrogenase (GAPDH), ERK, p-ERK, JNK, p-JNK, p38, p-p38, glucose-regulated protein 78 (GRP78), eukaryotic initiation factor 2 alpha (eIF2α), p-eIF2α, CCAAT/enhancer-binding protein homologous protein (CHOP; Thermo Fisher Scientific, Waltham, MA, USA), fibronectin (Abcam, Cambridge, MA, USA), transforming growth factor-β1 (TGF-β1; R&D Systems, Minneapolis, MN, USA), connective tissue growth factor (CTGF; Abcam, Cambridge, MA, USA), α-smooth muscle actin (α-SMA; Sigma-Aldrich, St. Louis, MO, USA), vimentin, E-cadherin (Abcam, Cambridge, MA, USA), Smad2/3, p-Smad2/3, NADPH oxidase 4 (NOX4; Novus Biologicals, Littleton, CO, USA), transferrin receptor 1 (TFR1; Abcam, Cambridge, MA, USA), glutathione peroxidase 4 (GPX4; Abcam, Cambridge, MA, USA), xCT (Proteintech, Rosement, IL, USA), p53, and Bax (Santa Cruz Biotechnology, Santa Cruz, CA, USA). Antibodies without specified suppliers were obtained from Cell Signaling Technology (Danvers, MA, USA). The membranes were then treated with HRP-conjugated secondary antibodies. The protein bands were detected using an enhanced chemiluminescence reagent, and band intensities were measured and analyzed with ImageJ software version 1.53j (National Institute of Health, Bethesda, MD, USA).

### 2.7. qRT-PCR

RNA was extracted from kidney tissues using TRIzol reagent, and cDNA was synthesized with the PrimeScript RT Reagent Kit (TaKaRa, Tokyo, Japan). PCR reactions were prepared with a mixture containing 100 ng of synthesized cDNA and 100 pM of each primer (Table 1). Amplification began with an initial denaturation phase at 95 °C for 10 min, followed by 45 cycles. Each cycle included heating the samples at 95 °C for 20 s to separate DNA strands, allowing the primers to bind at 60 °C for 30 s, and synthesizing new DNA strands at 72 °C for an additional 20 s. The qRT-PCR was carried out on the Thermal Cycler Dice Real-Time System III (TaKaRa, Tokyo, Japan) with SYBR Green. Relative gene expression was calculated using the 2^−ΔΔCT^ method, normalizing against *Actb* as the reference gene.

### 2.8. Statistical Analysis

Statistical analyses were conducted using GraphPad Prism version 8.3.0. (GraphPad Software, San Diego, CA, USA). Data are shown as the mean ± SEM. Normality of the data was assessed using the Shapiro–Wilk test, while variance homogeneity was analyzed using Bartlett’s test. For data that were normally distributed and exhibited homogeneous variances, a one-way ANOVA with Tukey’s multiple comparisons test was used to assess differences among groups. For data that were not normally distributed or did not meet the assumptions of homogeneity of variance, the Kruskal–Wallis test with Dunn’s multiple comparisons test was employed. A *p*-value below 0.05 was regarded as indicative of statistical significance.

## 3. Results

### 3.1. TA Ameliorates Renal Injury and Preserves Renal Function in a UUO-Induced CKD Model

**Figure 2:**
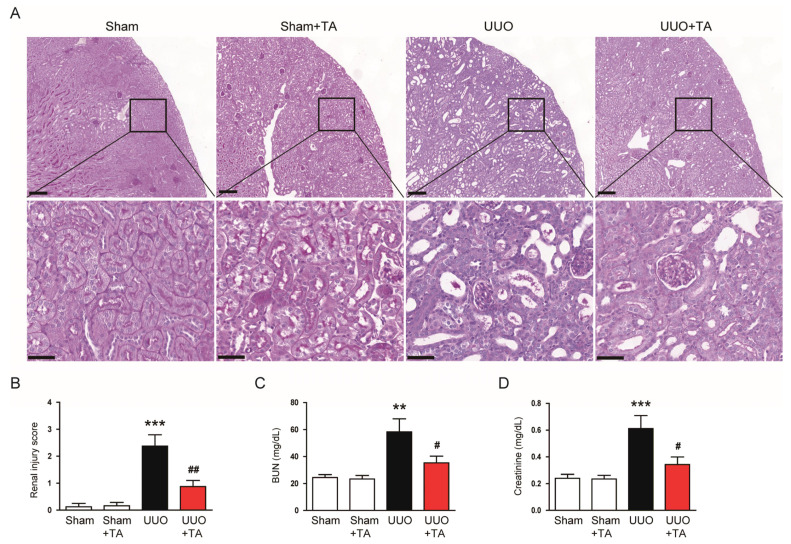
Impact of TA on histological damage and renal dysfunction in UUO mice. (**A**) PAS staining. Scale bar: 100 µm in the upper panel and 40 µm in the lower panel. (**B**) Tubular injury scores (n = 8). (**C**) Serum BUN concentrations (n = 8). (**D**) Serum creatinine concentrations (n = 8). Data are shown as mean ± SEM. ** *p* < 0.01 and *** *p* < 0.001 vs. sham. ^#^ *p* < 0.05 and ^##^ *p* < 0.01 vs. UUO.

In untreated UUO mice, the injured kidneys showed histological changes, such as tubular dilatation and cast formation (Figure 2A). However, these histopathological features were notably reduced in TA-treated mice (Figure 2A). This improvement was further demonstrated by a reduction in the renal injury score in the TA-treated group (Figure 2B). In addition to structural benefits, TA treatment also lowered serum BUN and creatinine levels (Figure 2C,D). Notably, TA alone showed no notable impact on renal structure (Figure 2A,B) or renal function (Figure 2C,D) in sham mice.

**Figure 3:**
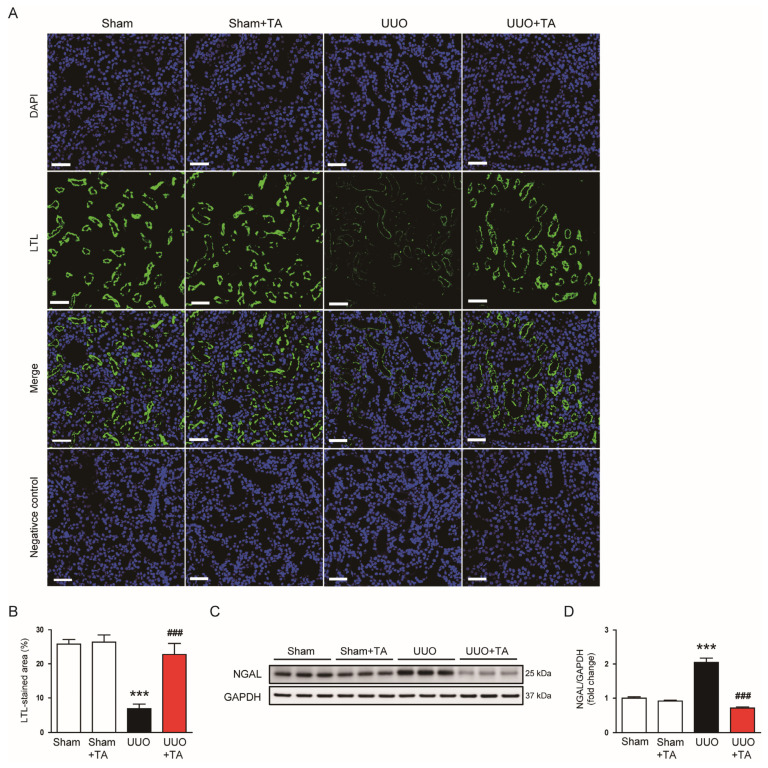
Impact of TA on proximal tubule brush border integrity and NGAL expression in mice with UUO-induced injury. (**A**) IF staining with fluorescently labeled LTL to assess brush border integrity. Scale bar: 50 μm. (**B**) Measurement of LTL-positive area as a percentage (n = 8). (**C**) Western blotting and (**D**) densitometric evaluation of NGAL (n = 6). Data are shown as mean ± SEM. *** *p* < 0.001 vs. sham. ^###^ *p* < 0.001 vs. UUO.

IF staining with fluorescently labeled LTL, specific to the proximal tubule brush border [22], was utilized to assess its integrity. The results showed a significantly smaller stained area in the UUO group compared to the sham group, indicating a pronounced loss of the brush border in the proximal tubules due to UUO-induced injury (Figure 3A,B). This reduction in LTL-positive staining emphasizes the extent of tubular damage caused by UUO. Notably, TA treatment markedly restored the LTL-stained area in UUO mice (Figure 3A,B). Furthermore, TA treatment significantly decreased the protein levels of NGAL, a widely used marker of tubular injury [23], in UUO mice (Figure 3C,D). This decrease in NGAL expression in TA-treated mice compared to untreated UUO mice highlights TA’s protective effects against tubular injury.

### 3.2. TA Attenuates Inflammatory Responses, MAP Kinase Pathway Activation, and ER Stress in UUO Mice

**Figure 4:**
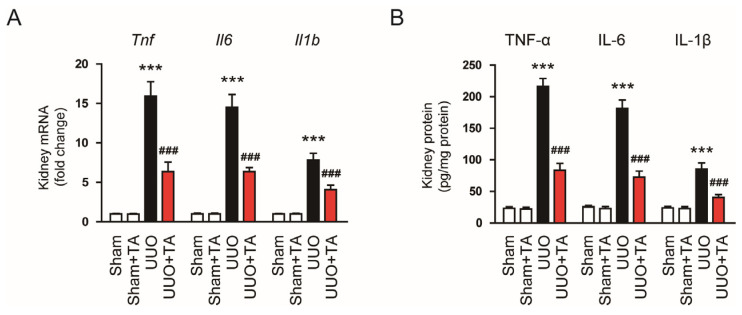
Impact of TA on cytokine expression in mice subjected to UUO. (**A**) *Tnf*, *Il6*, and *Il1b* mRNA levels (n = 8). (**B**) Protein levels of the same cytokines (n = 8). Data are shown as mean ± SEM. *** *p* < 0.001 vs. sham. ^###^ *p* < 0.001 vs. UUO.

The mRNA expression of *Tnf*, *Il6*, and *Il1b* was markedly elevated in the kidneys of UUO mice compared to controls (Figure 4A). The TA treatment notably lowered these mRNA levels (Figure 4A). Consistent with the mRNA data, their protein levels were also elevated in UUO kidneys (Figure 4B), but the TA treatment effectively suppressed these increases (Figure 4B).

**Figure 5:**
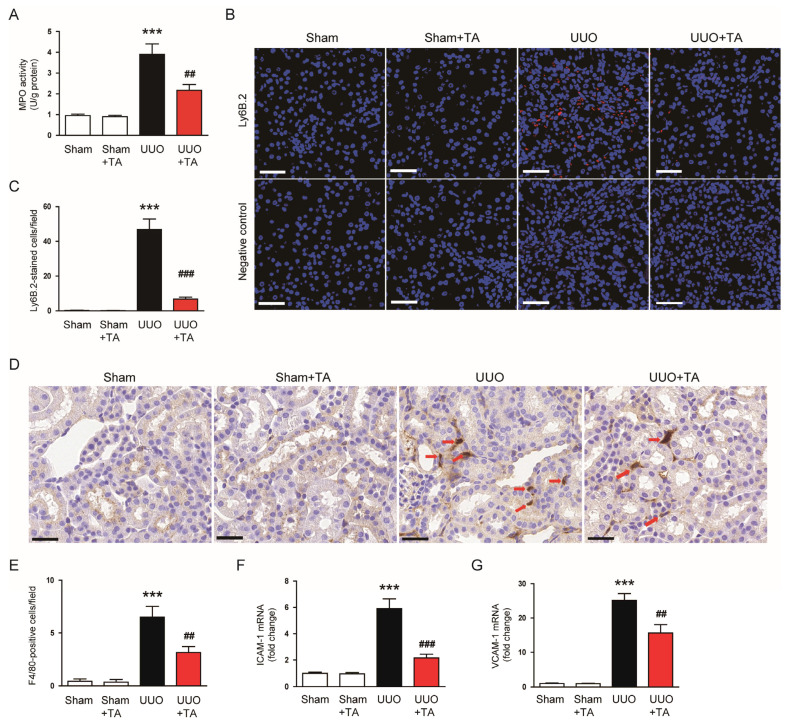
Impact of TA on immune cell accumulation in UUO mice. (**A**) MPO activities in renal tissue (n = 8). (**B**) IF staining for Ly6B.2. Scale bar: 50 µm. (**C**) Quantification of Ly6B.2-stained cells (n = 8). (**D**) IHC staining for F4/80. The red arrows indicate F4/80-positive cells. Scale bar: 60 µm. (**E**) Quantification of F4/80-stained cells (n = 8). (**F**) ICAM-1 mRNA levels (n = 8). (**G**) VCAM-1 mRNA levels (n = 8). Data are represented as mean ± SEM. *** *p* < 0.001 vs. sham. ^##^ *p* < 0.01 and ^###^ *p* < 0.001 vs. UUO.

MPO activity, a marker for neutrophil infiltration [24], was markedly increased in UUO kidneys (Figure 5A). The TA treatment significantly reduced MPO activity (Figure 5A). Furthermore, IF staining using an antibody against Ly6B.2, a neutrophil-specific marker [25], revealed a notable increase in neutrophils in UUO kidneys compared to controls (Figure 5B,C). The TA treatment markedly decreased the presence of Ly6B.2-expressing cells (Figure 5B,C). Elevated macrophage infiltration in UUO kidneys was observed, as indicated by F4/80 staining, a known macrophage marker [26] (Figure 5D,E). The TA treatment significantly decreased F4/80-positive cells (Figure 5D,E). Additionally, ICAM-1 and VCAM-1 mRNA levels were markedly upregulated in UUO kidneys (Figure 5F,G). The TA treatment effectively reduced the expression of both adhesion molecules (Figure 5F,G).

**Figure 6:**
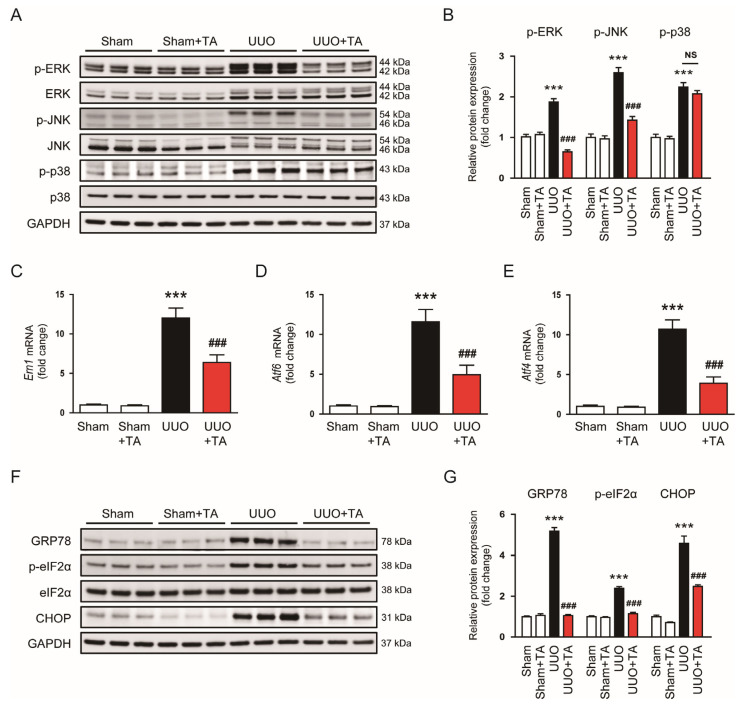
Impact of TA on MAP kinase and ER stress pathways in UUO mice. (**A**) Western blotting and (**B**) densitometric evaluation of phosphorylated forms of ERK, JNK, and p38 (n = 6). (**C**) *Ern1* mRNA levels (n = 8). (**D**) *Atf6* mRNA levels (n = 8). (**E**) *Atf4* mRNA levels (n = 8). (**F**) Western blotting and (**G**) densitometric evaluation of GRP78, p-eIF2α, and CHOP (n = 6). Data are presented as mean ± SEM. *** *p* < 0.001 vs. sham. ^###^ *p* < 0.001 vs. UUO. NS, not significant.

Given the established role of MAP kinase and ER stress pathways in promoting renal inflammation and tissue damage in CKD [27,28], we sought to evaluate the effects of TA on these pathways. In UUO kidney samples, phosphorylation levels of MAP kinase components ERK, JNK, and p38 were elevated (Figure 6A,B). The treatment with TA effectively reduced the phosphorylation of ERK and JNK (Figure 6A,B). However, the phosphorylation levels of p38 remained unchanged (Figure 6A,B).

To further assess the impact of TA on ER stress, we measured the mRNA expression levels of key ER stress markers, such as *Ern1*, *Atf6*, and *Atf4*. UUO kidneys showed an increased expression of these markers (Figure 6C–E). The TA treatment effectively lowered the mRNA levels of *Ern1*, *Atf6*, and *Atf4* (Figure 6C–E). The Western blot results further confirmed TA’s effect on ER stress at the protein level. UUO kidneys exhibited increased protein levels of GRP78, p-eIF2α, and CHOP, all critical mediators of the unfolded protein response and indicators of ER stress (Figure 6F,G). The TA treatment markedly reduced the expression of these ER stress markers (Figure 6F,G).

### 3.3. TA Reduces Fibrosis, Reverses EMT, and Inhibits Smad2/3 Activation in UUO-Induced Kidney Injury

**Figure 7:**
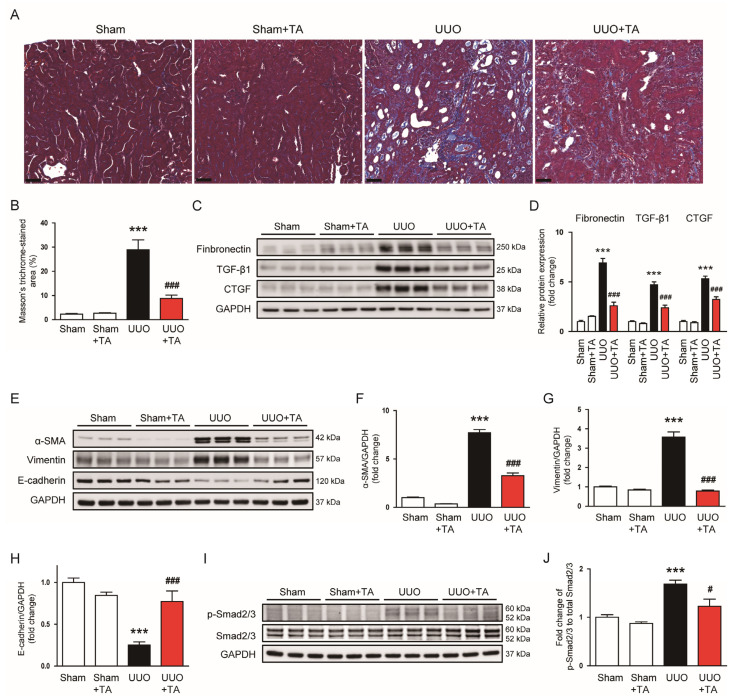
Impact of TA on renal fibrosis and EMT in the UUO-induced CKD model. (**A**) Masson’s trichrome staining. Scale bar: 50 μm. (**B**) Quantification of the fibrotic area (n = 8). (**C**) Western blotting and (**D**) densitometric evaluation of α-SMA, vimentin, and E-cadherin. (**E**) Western blotting and (**F**–**H**) densitometric evaluation of α-SMA, vimentin, and E-cadherin (n = 6). (**I**) Western blotting and (**J**) densitometric evaluation of p-Smad2/3 (n = 6). Data are presented as mean ± SEM. *** *p* < 0.001 vs. sham. ^#^ *p* < 0.05 and ^###^ *p* < 0.001 vs. UUO.

To assess the impact of TA on fibrosis in UUO mice, we analyzed indicators of fibrosis and epithelial-to-mesenchymal transition (EMT) in kidney tissues. Masson’s trichrome staining demonstrated a notable expansion of fibrotic regions in UUO kidneys (Figure 7A,B), indicating elevated collagen deposition and fibrosis. However, the TA treatment substantially reduced this stained area (Figure 7A,B). We then assessed the expression of key fibrotic markers. The protein expression of fibronectin, TGF-β1, and CTGF were largely elevated in UUO kidneys (Figure 7C,D), reflecting an increased fibrotic response. The TA treatment effectively reduced the expression of these fibrosis-associated proteins (Figure 7C,D).

Since EMT is a crucial process in fibrosis progression [29], we investigated the impact of TA on EMT markers. In UUO kidneys, the protein levels of α-SMA and vimentin, both markers associated with mesenchymal transition, was significantly increased, while E-cadherin, an epithelial marker, was decreased, indicating a shift towards EMT (Figure 7E–H). The treatment with TA reversed these changes, reducing α-SMA and vimentin levels while restoring E-cadherin expression (Figure 7E–H). Additionally, we explored the role of Smad2/3 activation, a key mediator of fibrosis and EMT [29]. The phosphorylation of Smad2/3 was significantly increased in UUO kidneys (Figure 7I,J). The TA treatment inhibited Smad2/3 phosphorylation (Figure 7I,J).

### 3.4. TA Alleviates Oxidative DNA Damage and Lipid Peroxidation in UUO-Induced Kidney Injury

**Figure 8:**
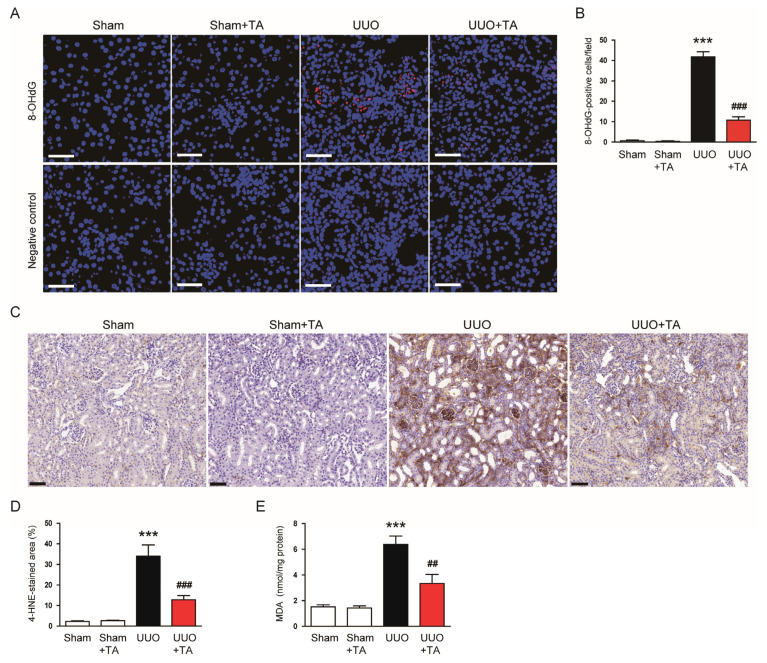
Impact of TA on oxidative DNA damage and lipid peroxidation in the UUO-induced CKD model. (**A**) IF staining for 8-OHdG. Scale bar: 50 μm. (**B**) Count of 8-OHdG-stained cells (n = 8). (**C**) IHC analysis for 4-HNE. Scale bar = 50 μm. (**D**) Percentages of 4-HNE-positive area (n = 8). (**E**) MDA concentrations in renal tissue (n = 8). Data are represented as mean ± SEM. *** *p* < 0.001 vs. sham. ^##^ *p* < 0.01 and ^###^ *p* < 0.001 vs. UUO.

Oxidative stress is a key contributor to CKD progression, as it causes cellular damage through ROS, promoting inflammation, fibrosis, and functional decline [2,3]. To evaluate the impact of TA on oxidative stress, we first examined oxidative DNA damage using 8-OHdG, an established indicator of DNA oxidation [30]. IF staining for 8-OHdG showed a marked rise in the number of 8-OHdG-stained cells in UUO kidneys (Figure 8A,B). TA treatment, however, markedly lowered the number of 8-OHdG-stained cells (Figure 8A,B).

Next, we assessed lipid peroxidation levels through 4-HNE staining, an established marker of lipid peroxidation [31]. The IHC results revealed a substantial expansion in the 4-HNE-positive staining area in UUO kidneys (Figure 8C,D). The TA treatment notably decreased this area (Figure 8C,D). Finally, we measured renal concentrations of MDA, another lipid peroxidation marker [31], to quantify oxidative stress. MDA levels were significantly elevated in UUO kidneys (Figure 8E), consistent with heightened lipid peroxidation. The TA treatment effectively reduced MDA levels (Figure 8E).

### 3.5. TA Inhibited Apoptosis and Ferroptosis in UUO-Induced Kidney Injury

**Figure 9:**
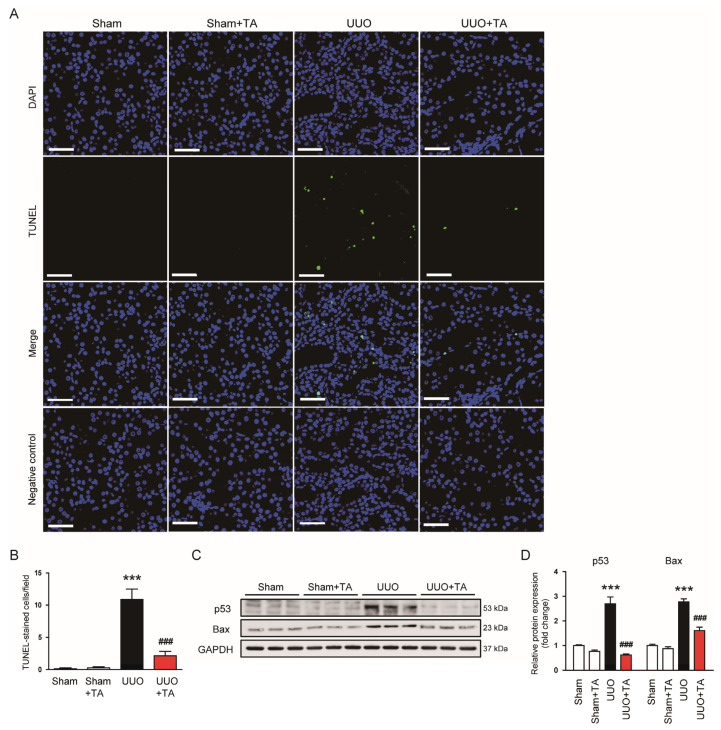
Impact of TA on apoptosis in UUO model. (**A**) TUNEL staining. Scale bar: 50 μm. (**B**) Count of TUNEL-labeled cells (n = 8). (**C**) Western blotting and (**D**) densitometric evaluation of p53 and Bax (n = 6). Data are presented as mean ± SEM. *** *p* < 0.001 vs. sham. ^###^ *p* < 0.001 vs. UUO.

Apoptosis and ferroptosis are crucial in CKD progression, as they contribute to cell death and tissue damage, which exacerbate renal dysfunction and fibrosis [5]. The TUNEL assay demonstrated a significant increase in apoptotic cells in UUO kidneys, which was markedly reduced by the TA treatment (Figure 9A,B). Additionally, the protein expression of apoptosis-related markers, p53 and Bax, were significantly increased in UUO kidneys, but the TA treatment reduced their expression (Figure 9C,D).

**Figure 10:**
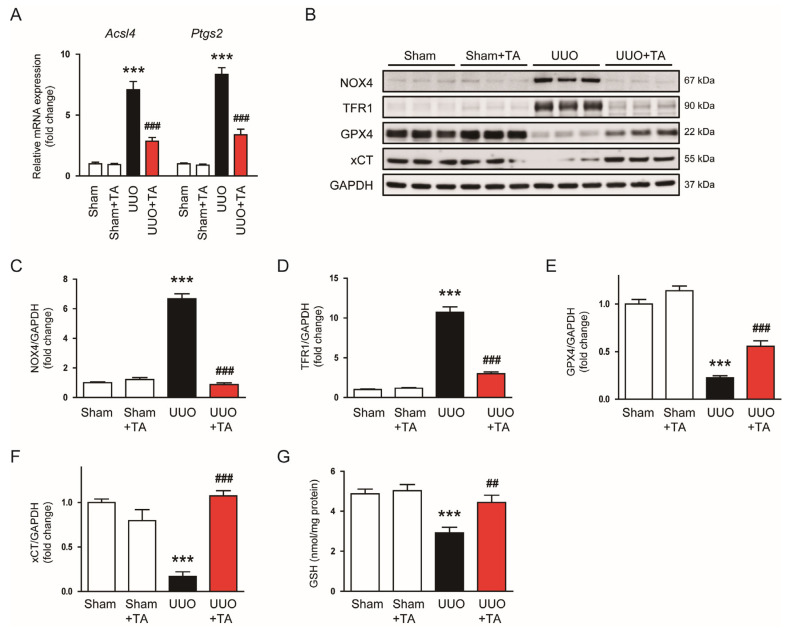
Impact of TA on ferroptosis in UUO kidneys. (**A**) mRNA levels of *Acsl4* and *Ptgs2* (n = 8). (**B**) Western blotting and (**C**–**F**) densitometric evaluation of NOX4, TFR1, GPX4, and xCT (n = 6). (**G**) GSH levels (n = 8). Data are represented as mean ± SEM. *** *p* < 0.001 vs. sham. ^##^ *p* < 0.01 and ^###^ *p* < 0.001 vs. UUO.

To assess the impact of TA on ferroptosis, we first analyzed the mRNA expression of ferroptosis markers *Acsl4* and *Ptgs2*. Both markers showed a notable elevation in mRNA levels in UUO kidneys (Figure 10A). The TA treatment lowered the mRNA expression of *Acsl4* and *Ptgs2* (Figure 10A). We further evaluated ferroptosis markers at the protein level. In UUO kidneys, the protein levels of NOX4 and TFR1, which promote ferroptosis, was elevated, while the expression of GPX4 and xCT, known inhibitors of ferroptosis, was decreased (Figure 10B–F). The TA treatment reversed these changes, decreasing NOX4 and TFR1 levels while restoring GPX4 and xCT levels (Figure 10B–F). Furthermore, changes in xCT expression were associated with alterations in GSH levels, an important antioxidant involved in preventing ferroptosis. GSH levels were significantly decreased in UUO kidneys (Figure 10G) However, the TA treatment restored GSH levels (Figure 10G), which aligns with the recovery of xCT expression.

## 4. Discussion

This study investigated the protective effects of TA on UUO-evoked kidney injury, focusing on its potential to alleviate key pathological processes in CKD, such as inflammation, fibrosis, oxidative stress, apoptosis, and ferroptosis. Recognizing the role of tubular cell death in exacerbating renal inflammation and fibrosis [32], we assessed the impact of TA on these processes. Notably, to the best of our knowledge, this is the first study to demonstrate TA’s multifaceted renoprotective effects through its action on critical pathogenic pathways in CKD.

TA exhibited significant protective effects on renal injury, tubular integrity, and renal function in UUO-induced kidney damage. The reduction in the renal injury score in TA-treated mice compared to untreated UUO mice highlights TA’s overall protective impact on renal tissue architecture. This decrease in the injury score correlates with improvements in tubular structure, as evidenced by the restoration of the brush border integrity in proximal tubules. In UUO kidneys, LTL staining demonstrated a loss of the brush border structure, indicative of severe tubular damage. However, the TA treatment markedly restored the LTL-positive area, suggesting its role in preserving tubular integrity. Furthermore, TA significantly reduced the expression of NGAL, a biomarker of tubular injury that is elevated in response to renal damage [33,34]. The decrease in NGAL expression in TA-treated kidneys indicates a reduction in tubular injury, underscoring TA’s protective effects on tubular cells. Beyond structural improvements, TA also lowered serum levels of BUN and creatinine, two key markers of renal function. The reduction in these biomarkers suggests that TA not only mitigates structural kidney damage but also helps preserve renal function in UUO-induced kidney injury, indicating its potential to slow CKD progression by safeguarding renal function.

This study shows that TA treatment effectively reduces the production of pro-inflammatory cytokines, indicating strong anti-inflammatory effects. Additionally, TA decreases immune cell infiltration, including neutrophils and macrophages, by downregulating adhesion molecules, which likely protects renal tissue from inflammation-induced damage. These findings suggest TA’s potential as a therapeutic agent for alleviating kidney inflammation in CKD. In addition to reducing cytokine production and immune cell infiltration, TA modulates the MAP kinase signaling cascade, a key regulator of inflammatory and stress responses in CKD [35]. Our findings demonstrate that the TA treatment decreases the phosphorylation levels of ERK and JNK, two major components of the MAP kinase pathway, suggesting that TA inhibits the activation of these kinases in response to UUO-induced injury. However, TA had no effect on p38 phosphorylation, indicating its selective action within the MAP kinase family. This selective inhibition could imply a targeted therapeutic effect, potentially reducing specific inflammatory responses without affecting the entire signaling network, which may minimize unintended side effects. Lastly, TA’s impact on ER stress, a critical contributor to CKD pathology [36,37,38], is substantial. The TA treatment effectively lowered both mRNA and protein levels of several ER stress markers, including IRE1α, ATF6, ATF4, GRP78, p-eIF2α, and CHOP. The attenuation of these markers, particularly CHOP, which is closely linked to cell apoptosis during prolonged ER stress [39,40], suggests that TA may alleviate ER stress-evoked cell death. This suppression of ER stress aligns with TA’s reduction of inflammation, as ER stress is known to exacerbate inflammatory responses in kidney injury [41,42]. By modulating ER stress and selectively inhibiting specific MAP kinase components, TA provides a dual protective effect against inflammatory and stress-induced pathways, thereby offering a potentially effective strategy for mitigating kidney injury in CKD.

One of the significant findings of this study is the ability of TA to mitigate renal fibrosis. We observed that the TA treatment significantly reduced fibrotic markers such as fibronectin, TGF-β1, and CTGF, as well as key EMT markers α-SMA and vimentin. Furthermore, TA increased E-cadherin levels, a marker of epithelial cells, indicating its role in preserving tubular integrity. By inhibiting the phosphorylation of Smad2/3, a downstream effector of the TGF-β signaling cascade [43], TA disrupts a key fibrotic mechanism, ultimately limiting extracellular matrix (ECM) deposition. These results suggest that TA effectively attenuates fibrosis by targeting both fibrotic signaling pathways and EMT. EMT is essential for the transformation of tubular cells into myofibroblasts, which are the primary source of ECM in CKD [43]. Supporting evidence for TA’s anti-fibrotic effects has also been demonstrated in hepatic fibrosis, where TA reduced liver fibrosis by modulating multiple pathways [44]. TA’s action decreased collagen deposition, hepatocyte apoptosis, and altered gene expression linked to fibrosis, supporting its tissue-protective properties. The results of the hepatic fibrosis study reinforce our findings in renal fibrosis, where TA’s inhibition of the TGF-β/Smad2/3 cascade parallels its effects on other fibrotic pathways observed in liver fibrosis. By targeting these interconnected signaling pathways, TA appears to exert comprehensive anti-fibrotic effects across different organ systems. Thus, our study contributes to the growing body of evidence highlighting TA’s broad anti-fibrotic potential by demonstrating its effectiveness in limiting ECM deposition and EMT in CKD.

Our study also highlights the role of oxidative stress in CKD pathophysiology and TA’s capacity to reduce this damaging factor. Oxidative stress not only promotes inflammation and fibroblast activation but also contributes to cellular apoptosis and ferroptosis, thereby amplifying renal damage [45]. The TA treatment significantly reduced markers of lipid peroxidation, such as 4-HNE and MDA, as well as the oxidative DNA damage marker 8-OHdG, indicating an overall reduction in oxidative stress. Importantly, TA modulated key redox enzymes, including reducing the prooxidant enzyme NOX4 and restoring antioxidant defenses such as GPX4 and xCT, which are critical for ferroptosis resistance [46,47,48]. By improving GSH levels, TA further bolstered antioxidant defenses, which aligns with the restoration of xCT expression, a component of the cystine/glutamate antiporter system necessary for GSH synthesis. This antioxidative action of TA is essential for mitigating oxidative stress and its downstream effects on inflammation and fibrosis.

In CKD, both apoptosis and ferroptosis play pivotal roles in driving renal inflammation and fibrosis, significantly contributing to disease progression [49,50,51]. Apoptosis, often triggered by oxidative stress, leads to the loss of tubular cells, which subsequently worsens inflammation and fibrosis in kidney tissues [49]. This process is marked by the release of pro-inflammatory signals from dying tubular cells, attracting immune cells and intensifying inflammatory responses, which in turn activate fibrotic pathways. This self-sustaining cycle of oxidative stress, inflammation, and cell death underlies much of the progressive tissue damage observed in CKD [49]. Ferroptosis is particularly relevant in CKD due to dysregulated iron metabolism and excessive lipid peroxidation—both common features in CKD pathology [52,53]. In renal cells, ferroptosis can lead to significant tubular injury and release lipid peroxidation products that stimulate further inflammation and fibrosis [9,10]. The iron overload often present in CKD patients increases susceptibility to ferroptosis, which not only damages kidney cells but also amplifies oxidative stress within the renal microenvironment, worsening the disease mechanisms [54]. Thus, inhibiting both apoptosis and ferroptosis is essential to disrupting this cycle of cell death and tissue damage, offering a targeted approach to slowing CKD progression.

Our study demonstrates that TA has a promising dual inhibitory effect on both apoptosis and ferroptosis in UUO-induced kidney injury. TA diminished the presence of TUNEL-labeled apoptotic cells and lowered the expression of critical apoptotic markers, p53 and Bax. By mitigating apoptosis, TA helps prevent tubular cell loss, subsequently reducing inflammation and fibrosis. This effect is likely linked to TA’s antioxidant properties, as oxidative stress is a major trigger of apoptosis in kidney injury. By reducing oxidative stress, TA may indirectly limit apoptosis initiation, thereby preserving tubular cell integrity and function. Furthermore, TA exhibited anti-ferroptotic effects in UUO-induced kidney injury. NOX4, a key source of ROS in the kidney [55,56], contributes to lipid peroxidation and drives ferroptosis. By downregulating NOX4, TA likely reduces ROS production, thereby limiting lipid peroxidation and ferroptosis in renal cells. Additionally, the modulation of ferroptosis-related proteins like GPX4 and xCT, which are central to cellular antioxidant defenses, further suggests that TA helps restore redox balance in the kidney. GPX4, for instance, is critical in protecting cells from lipid peroxidation [46,47]. Its modulation by TA indicates that TA supports ferroptotic resistance, enhancing cellular defenses against lipid-induced damage. The antioxidant properties of TA may thus be a key factor in its ability to inhibit both apoptosis and ferroptosis. By attenuating oxidative stress, TA not only prevents the activation of apoptosis pathways but also reduces the oxidative conditions necessary for ferroptosis. This dual action positions TA as a multifaceted protective agent capable of interrupting the interconnected processes of inflammation, fibrosis, apoptosis, and ferroptosis in CKD.

This study highlights the protective effects of TA in a UUO-induced CKD model, demonstrating its ability to mitigate key pathological processes such as inflammation, fibrosis, oxidative stress, apoptosis, and ferroptosis. While these findings emphasize the potential of TA in addressing CKD, several limitations need to be considered for a balanced interpretation. Although direct measurements of ROS were not performed, validated indirect markers such as 8-OHdG, 4-HNE, and MDA were used to provide a comprehensive assessment. Incorporating direct ROS measurements in future studies would offer further insights into the underlying mechanisms. Additionally, this study utilized a UUO mouse model, which replicates some features of CKD but does not fully capture the complexity of human CKD pathology, thereby limiting the direct applicability of these findings to clinical settings. While renal function was assessed using serum biomarkers such as BUN and creatinine, urinary markers like proteinuria were not included due to logistical constraints, which future research could address to provide a more complete evaluation of renal function. Another limitation lies in the use of GAPDH for normalization in oxidative stress-related experiments; while widely used, its expression can vary under oxidative stress. Future studies will implement alternative normalization methods, such as total protein normalization, to improve the reliability of the data. Finally, this study employed a single dose of TA, which was effective in reducing kidney injury in the UUO model. However, dose–response studies are needed to determine the most effective and safe dosage. Addressing these limitations in future research will deepen our understanding of TA’s mechanisms and its potential role in managing CKD.

## 5. Conclusions

In summary, this study demonstrates that TA exerts comprehensive protective effects in UUO-induced renal injury by targeting multiple pathological processes, including oxidative stress, apoptosis, and ferroptosis. By modulating these pathways, TA reduces inflammatory responses and mitigates fibrotic progression. These findings highlight TA’s potential as an effective intervention for CKD, providing a basis for further research to elucidate its clinical applicability in managing CKD and renal fibrosis.

## Figures and Tables

**Table 1:** List of primers.

Gene	Primer Sequence (5′→3′)	Accession No.
*Tnf*	F: ACTTCGGGGTGATCGGTCCCC R: TGGTTTGCTACGACGTGGGCTAC	NM_013693
*Il6*	F: TACCACTTCACAAGTCGGAGGC R: CTGCAAGTGCATCATCGTTGTTC	NM_031168
*Il1b*	F: CGCAGCAGCACATCAACAAGAGC R: TGTCCTCATCCTGGAAGGTCCACG	NM_008361
*Icam1*	F: AACTGTGGCACCGTGCAGTC R: AGGGTGAGGTCCTTGCCTACTTG	NM_010493
*Vcam1*	F: CCCAGGTGGAGGTCTACTCA R: CAGGATTTTGGGAGCTGGTA	NM_011693
*Ern1*	F: GGTCCAATCGTACGGCAGTT R: TCTCTCACAGAGCCACCTTTGTAG	NM_023913
*Atf6*	F: CCCAAGCTCTCCGCATAGTC R: TAAAATGCCCCATAACTGACCAA	NM_001081304
*Atf4*	F: GAGCTTCCTGAACAGCGAAGTG R: TGGCCACCTCCAGATAGTCATC	NM_009716
*Acsl4*	F: CGTTCCTCCAAGTAGACCAAC R: CCTTACACTGTCTGACCAGTC	NM_207625
*Ptgs2*	F: TGCCTGGTCTGATGATGTATG R: GCCCTTCACGTTATTGCAGATG	NM_011198
*Actb*	F: GGCTGTATTCCCCTCCATCG R: CCAGTTGGTAACAATGCCATGT	NM_007393

## Data Availability

All data analyzed during the current study are included within this article.
